# Prednisolone versus antihistamine for allergic rhinitis: No significant difference found in randomized trial

**DOI:** 10.1002/clt2.70017

**Published:** 2025-01-09

**Authors:** Carl Skröder, Laila Hellkvist, Ulla Westin, Pernilla Sahlstrand‐Johnsson, Kerstin Hansson, Agneta Karlsson, Åslög Dahl, Leif Bjermer, Lars Olaf Cardell

**Affiliations:** ^1^ Department of Otorhinolaryngology, Head & Neck Surgery Skane University Hospital Lund Sweden; ^2^ Division of ENT Diseases Department of Clinical Sciences, Intervention and Technology Karolinska Institutet Stockholm Sweden; ^3^ Department of ENT Diseases Karolinska University Hospital Stockholm Huddinge Sweden; ^4^ Department of Respiratory Medicine and Allergology Lund University Skane University Hospital Lund Sweden; ^5^ Departments of Biological and Environmental Sciences Gothenburg University Gothenburg Sweden

**Keywords:** allergic rhinitis, antihistamines prednisolone, randomized trial, treatment efficacy

## Abstract

**Background:**

Seasonal allergic rhinitis (AR) impacts public health by affecting work productivity and quality of life. The Swedish tree pollen season starts in February with alder and hazel pollination, followed by birch and ends with oak in May. Systemic corticosteroids are often prescribed when topical treatments fail, despite limited evidence supporting their efficacy.

**Objective:**

To compare the effectiveness of prednisolone tablets versus antihistamine tablets in reducing symptoms and medication usage in patients with moderate to severe tree pollen‐induced AR.

**Methods:**

This interventional single‐center, double‐blinded randomized trial included 34 patients. Treatment was initiated, and symptoms were registered during the tree pollen season. The two groups received either prednisolone tablets (20 mg) or ebastine tablets (20 mg) for 7 days. Treatment effects were evaluated by comparing daily symptom scores, use of topical medication, and a combined symptom‐medical score between the groups. Quality of life was recorded at the start and after 3 weeks.

**Results:**

Both interventions demonstrated efficacy in enhancing quality of life metrics. The area under the curve (AUC) for the combined symptom severity and medication usage score averaged 34.0 (SD = 19.1, 95% CI = 24.5–43.4) in the group treated with prednisolone. This was marginally lower than the control group, with an AUC of 32.6 (SD = 13.2, 95% CI = 25.6–39.7). The difference was not statistically significant (*p* = 0.80). Both groups exhibited only mild adverse events, which were statistically comparable in frequency and severity.

**Conclusions:**

Prednisolone tablets did not show superior efficacy over antihistamine tablets in reducing symptoms or medication usage in tree pollen‐induced AR. These results suggest that systemic corticosteroids may not provide additional benefits over antihistamines, and clinicians should prioritize individualized treatment based on patient preferences and tolerability.

## 1 INTRODUCTION

Allergic rhinitis (AR) is prevalent, affecting up to a third of the Swedish population,^1^ particular those in working age. AR is characterized by symptoms such as sneezing, nasal congestion, and ocular irritation, significantly impairing the quality of life and contributing to productivity losses in society.^1^, ^2^, ^3^

Current guidelines recommend systemic corticosteroids as a secondary treatment option when topical therapies fail; however, evidence supporting their efficacy is predominantly derived from outdated trials and clinical experience, yielding a low level of evidence.^2^ Variances in the administration and dosing of systemic corticosteroids among physicians underscore the need for further investigation into optimal treatment regimens.^2^, ^4^

Most studies investigating the effect of systemic steroids on treating AR were predominantly published in the late 80s.^5^, ^6^ A study by Brooks and colleagues in 1993 compared various doses of oral methylprednisolone, three times daily, during the first 4 days of the rag weed season.^7^ Another study from 2013 compared intranasal steroid spray (mometasone furoate) with betamethasone oral tablets in an open label protocol, both suggesting potential benefits of systemic steroids.^8^

In 2023, our group conducted a study comparing intramuscular methylprednisolone with placebo as treatment for AR during the birch pollen season of 2019.^9^ We observed a significant reduction in symptoms and use of topical treatment among patients who received systemic corticosteroids, although the clinical relevance of this effect was uncertain.^9^ Our results do not significantly differ from some other considerably older studies that do not meet modern study guidelines.^10^

The present study aimed to examine the comparative efficacy of prednisolone tablets versus antihistamine tablets in treating AR, given the anecdotal reports of positive effects of systemic corticosteroids in clinical practice. Our primary hypothesis posited that a short course of prednisolone tablets would significantly alleviate AR symptoms during the birch pollen season.

## 2 METHODS

### 2.1 Study design

This interventional single‐center double‐blinded randomized trial consisted of two parallel groups: 18 participants in the treated group and 16 in the control group.

**FIGURE 1:**
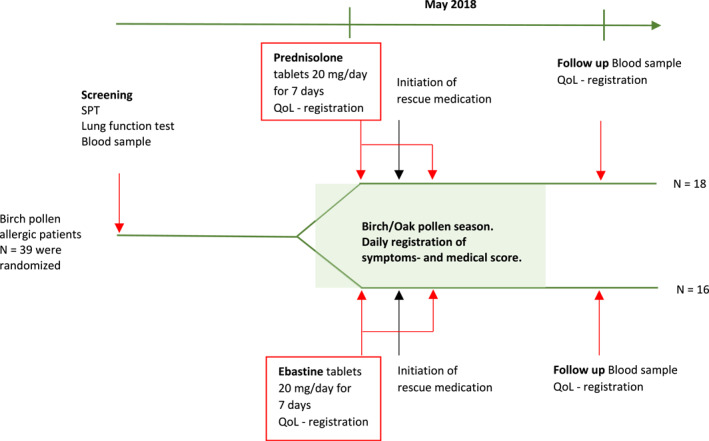
Flow chart. Green area represents the tree pollen season. Red arrows indicate visits and treatment start/stop.

Participants in the treated group received Prednisolone tablets, while those in the control group were administered ebastine tablets for 7 days. Symptoms were recorded daily, and quality‐of‐life questionnaires (Sinonasal Outcome Test 22 (SNOT‐22) and Juniper RQLQ) were completed at the beginning and end of the 3‐week trial period (Figure 1).

### 2.2 Study subjects

Individuals aged 22–38 with verified seasonal moderate to severe rhinoconjunctivitis during the birch season were eligible. Exclusion criteria were upper respiratory disease (non‐allergic sinusitis, nasal polyposis, chronic obstructive and restrictive lung disease), known autoimmune or collagen disease, renal disease, cancer, hepatic disease, cardiovascular disease, medication with a possible side‐effect of interfering with the immune response, previous immuno‐ or chemotherapy, major metabolic disease, alcohol or drug abuse, mental incapability of coping with the study, suspicion of or confirmed bacterial infection, pregnancy, wish for pregnancy, nursing or withdrawn informed consent.

### 2.3 Treatment protocol

Participants were allocated to treatment arms using a computer randomization program in blocks. Both participants and investigators were blinded to treatment allocation.

Participants in the active treatment group received prednisolone 20 mg, whereas those in the control group were administered ebastine 20 mg. To ensure blinding, all tablets were coated using Medcoat, rendering them indistinguishable in taste, size, and form. Throughout the initial week of the trial (Day 1–7), the research nurse personally dispensed the medication once daily to each participant.

Prior to the study's commencement, participants received a “Rescue medication package” containing Desloratadine tablets (5 mg *×* 1), Sodium cromoglycate eye drops (40 mg/mL, 1–2 drops *×* 2), and Mometasone Furoate nasal spray (50 μm *×* 2). However, the use of mometasone nasal spray and cromoglycate eye drops was restricted until Day 3 after experiencing symptoms for two consecutive days, after which they were permitted throughout the trial. Similarly, the use of desloratadine tablets was authorized after Day 7 and could be continued throughout the trial period.

### 2.4 Ethical approval

The study was approved by the local ethics committee in Lund, Sweden (Dnr: 2017/947) and registered in the EudraCT database (no. 2017‐004435‐37). All procedures adhered to good clinical practice, national guidelines, and the Declaration of Helsinki, with all participants providing written informed consent.

### 2.5 Outcome measures

The primary objective was to assess the reduction in symptoms and the usage of topical medication (standard of care) in patients treated with prednisolone tablets compared to those receiving standard antihistamine tablets^11^ Daily symptoms and medication usage were recorded using the RedCap software and quantified as a daily symptom score (dSS), daily medication score (dMS), and daily combined symptom‐ and medication score (dCSMS) following the EAACI's recommended scoring system.^11^ Symptoms such as runny nose, itchy nose, sneezing, blocked nose, red eyes, and itchy eyes were graded on a scale of 0–3, and a daily mean was calculated. Participants were instructed to utilize rescue medications incrementally. The daily medication score (dMS) ranged from 0 to 2, with no use of rescue medication assigned 0, use of topical/oral antihistamines assigned 1, and use of nasal steroids assigned 2. As systemic steroids other than the study drug were excluded, the maximum dMS value in this study was 2. Daily CSMS was derived by summing dSS and dMS, resulting in a score ranging from 0 to 5. The median values of dSS, dMS, and dCSMS for each group were computed daily throughout the study period. Differences between the two groups were expressed as the area under the curve (AUC). Although a minimal clinically important difference (MCID) for combined symptom‐ and medication score has yet to be established, we estimated a significant MCID to be approximately 20%.^12^

Secondary outcomes included the evaluation of quality of life using the (SNOT‐22) and the Juniper Rhinoconjunctivitis Quality of Life questionnaire at trial initiation and three weeks post‐treatment.^13^ The SNOT‐22, originally designed to assess chronic rhinosinusitis, comprises 22 questions graded on a scale of 0–5. Scores ranging from 8 to 20 indicate a mild impact on quality of life, while scores exceeding 20 suggest moderate to severe impacts. Although the use of SNOT‐22 for evaluating AR has not been fully validated, preliminary studies have indicated promising results, with a suggested MCID between 6 and 11.^14^

The Juniper RQLQ consists of seven sections with 28 questions graded from 0 to 6, and the mean total score was presented with an MCID of 0.5.^15^

Changes in the bone turnover marker (CTX) were analyzed both before and after the trial to evaluate any potential effects of the treatment.

#### 2.5.1 Pollen data, spirometry, skin prick test, and blood measurement

Pollen concentrations were measured using a Burkard 7‐day Volumetric Spore Trap situated at Skåne University Hospital, Malmö.

Spirometry using Care fusion (Höchberg, Germany) equipment was conducted to assess normal lung function in all participants before the commencement of the trial.

The presence of birch pollen allergy was verified through a skin prick test (SPT) utilizing the SPT ALK Soluprick® method. A wheal reaction of ≥3 mm was considered indicative of a positive test.

Venous blood samples were obtained from all participants during the screening visit and approximately 4 to 5 weeks after the initiation of the trial for comprehensive blood measurements. Elevated levels of specific IgE toward birch and oak were confirmed in all patients.

### 2.6 Statistical analysis and sample size

dSS, dMS, and dCSMS were quantified as the AUC, and intergroup differences were assessed using the Mann–Whitney test. Additionally, a Wilcoxon matched‐pairs signed rank test was employed to analyze changes in SNOT‐22 and RQLQ scores between day 1 and day 21 within each group. Intergroup differences in individual changes from day 1 to day 21 were evaluated using the Mann–Whitney test. A time‐series analysis was conducted using a generalized additive model (GAM) to assess the relative risk (RR) for symptoms in the study group over time.

Sample size calculation was based on a two‐sample *t*‐test, assuming equal sample sizes and variances, to detect an expected improvement in the rhinitis total symptom score of 2 out of 12 points. With a targeted power of 0.80 and a type 1 error rate (*α* level) of 0.05, the calculated sample size was 32. A power calculation using the CSMS was not conducted prior to the study. Notably, patients treated with Prednisolone and antihistamine had a median (interquartile range) CSMS score of 1.6 (1.0–1.9) and 1.7 (0.9–2.1), respectively.

Missing values were handled using the last observation carried forward technique. The statistical analyses were performed using GraphPad Prism 6.01 software (GraphPad Software).

## 3 RESULTS

### 3.1 Patients

**FIGURE 2:**
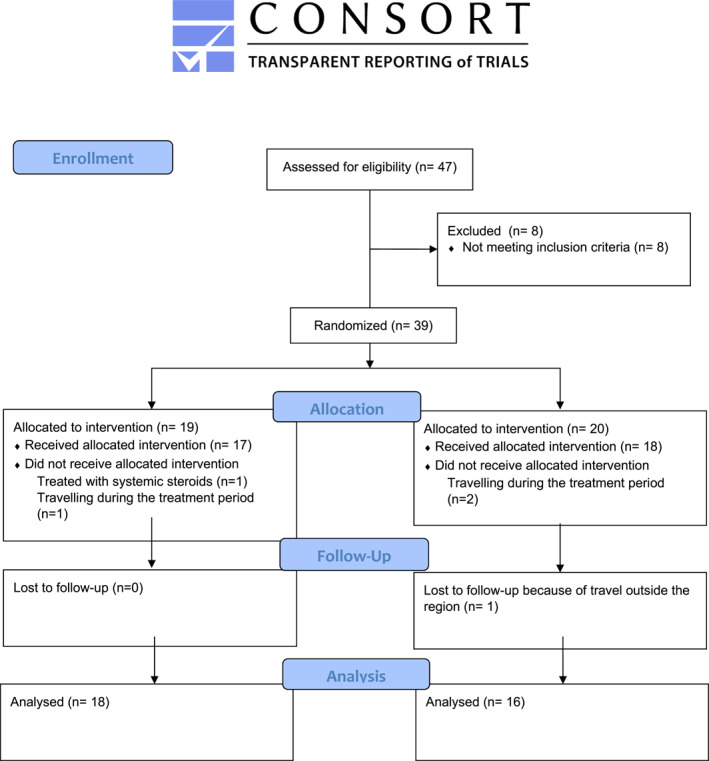
Consort 2010 flow diagram.

Thirty‐nine patients were randomized into two groups, 19 in the active group and 20 in the control group (Figure 2, CONSORT flow diagram). Demographics and baseline characteristics were similar between the groups, except for a higher frequency of other allergies (furry animals, grass, and mug worth) in the active group (Table 1). Both groups exhibited an equal response rate (>94%). Missing data of 4 days or less were accepted, and when calculating the AUC, missing data were extrapolated from the previous day using the “last‐object‐carried‐forward” principle, primarily due to travel reasons, with none registered.

**TABLE 1:** Background characteristics.

	Control group	Active group	*p*‐value
Number of patients	16	18	
Gender			
Female, no. (%)	8 (50%)	9 (50%)	
Male, no. (%)	8 (50%)	9 (50%)	
Age, mean (SD [range])	29 (5[22–37])	30 (4[22–38])	0.51
Sensitization to birch pollen on SPT, no. (%)	15 (94%)	18 (100%)	
Birch specific IgE (kU/L), median (SD [range])	24.1 (25.3 [4.2–100.0])	26.2 (19.3 [2.12–66.4])	0.79
Other allergy			
House dust mite, no. (%)	4 (25%)	5 (28%)	
Gras, no. (%)	8 (50%)	14 (78%)	
Mugwort, no. (%)	4 (25%)	10 (56%)	
Furry animals, no. (%)	5 (31%)	12 (67%)	
Verified seasonal asthma, no. (%)	4 (25%)	4 (22%)	
Use of beta‐2‐agonist, no. (%)	4 (25%)	3 (17%)	
Use of inhaled corticosteroids, no. (%)	1 (6%)	2 (11%)	

### 3.2 Symptom‐ and medication scores

**FIGURE 3:**
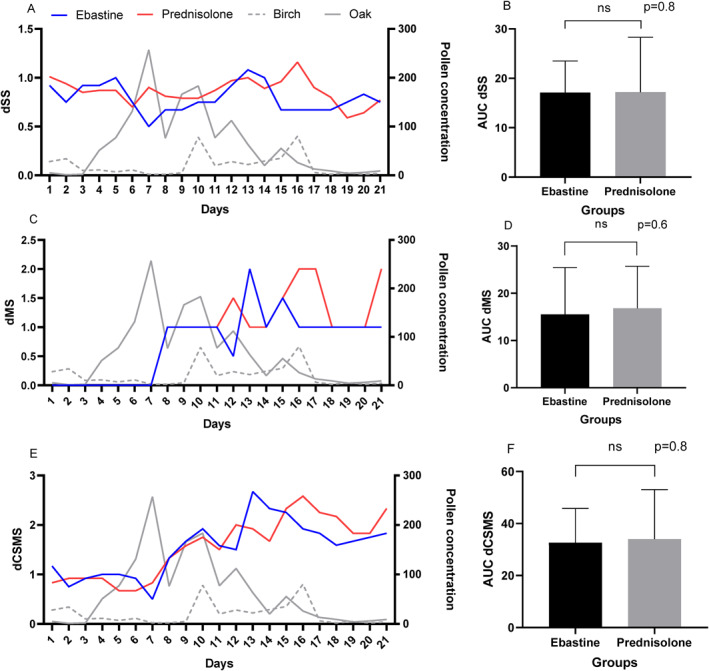
Panels (A, C, E) represent the daily median scores (dSS, dMS and dCSMS) in each group from day 1–21. Panels (B, D, F) shows the mean of total AUC (dSS, dMS and sCSMS) in each group. *p*‐value from Mann–Whitney test. AUC, area under the curve; dCSMS, daily combined symptom‐ and medication score; dMS, daily medication score; dSS, daily symptom score.

During the end phase of the 2018 tree pollen season, no significant difference was observed in dSS, dMS, and dCSMS between the two groups. The mean AUC for symptoms was 17.2 (SD 11.1 [95% CI 11.7–22.7]) in the treated group compared to 17.2 (SD 6.4 [95% CI 13.7–20.5]) in the control group, with *p* = 0.83. There was also no significant difference in the use of rescue medication, with mean AUCs of 16.8 (SD 8.9 [95% CI 12.3–21.2]) in the treated group compared to 15.5 (SD 9.9 [95% CI 10.2–20.8]) in the control group, with *p* = 0.60. The mean AUC of the combined symptom and medical score was 34.0 (SD 19.1 [95% CI 24.5–43.4]) in the prednisolone‐treated group and approximately 4% lower in the control group, 32.6 (SD 13.2 [95% CI 25.6–39.7]), with *p* = 0.80 (Figure 3).

### 3.3 Pollen data analysis

**FIGURE 4:**
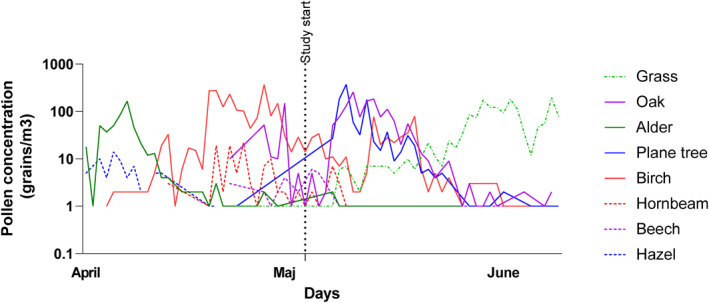
Pollen count during the tree pollen season of 2018 (Logarithmic scale).

During the observation period, the levels of birch pollen were found to be lower (with a seasonal pollen index [SPI] of 4959) compared to the historical average for the season (SPI 2501 between 1975 and 2018). Conversely, oak pollen levels were higher (with an SPI of 1615) compared to the average seasonal levels observed between 1975 and 2018 (SPI 1165). The study commenced toward the conclusion of the birch pollen season but prior to the peak of the oak pollen season (refer to Figures 4 and S1).

**FIGURE 5:**
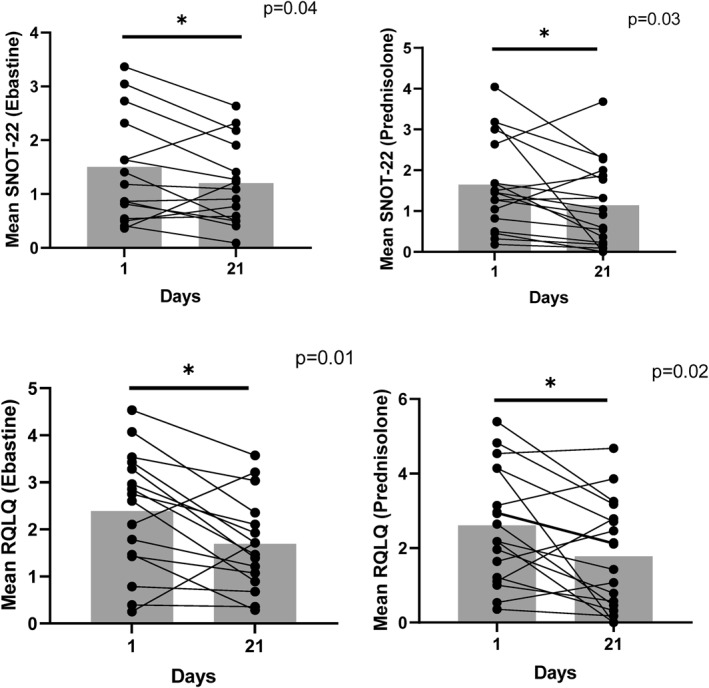
The difference in quality of life (Juniper RQLQ questionnaire and SNOT‐22 questionnaire) at day 1‐21 between each group. *p*‐value from Wilcoxon matched‐pairs signed rank test.

Birch pollen levels were lower (SPI 4959) than the average season (SPI 2501 between 1975 and 2018), whereas oak pollen levels were higher (SPI 1615 compared to the average season between 1975 and 2018 SPI 1165). The study period started at the beginning of the birch pollen season and before the oak pollen peak of the season (Figure 5 and Figure S1).

### 3.4 Quality of life

Both the treated and control groups exhibited improvements in quality of life from day 1 to 21. The mean SNOT‐22 score in the treated group decreased from 1.65 (SD 1.12, 95% CI 1.09–2.21) on day 1 to 1.14 (SD 1.02, 95% CI 0.63–1.65) on day 21 with a *p*‐value of 0.03. In comparison, the control group's mean SNOT‐22 score decreased from 1.50 (SD 0.98, 95% CI 0.98–2.02) on day 1 to 1.20 (SD 0.75, 95% CI 0.80–1.60) on day 21 with a *p*‐value of 0.04. The mean total SNOT‐22 scores for both groups were moderate at the start of the trial (33.0 for the control group and 36.3 for the prednisolone‐treated group) and showed improvement after 3 weeks (26.5 for the control group and 25.1 for the prednisolone‐treated group).^13^

Similarly, the mean Juniper RQLQ score in the treated group decreased from 2.61 (SD 1.52, 95% CI 1.85–3.36) on day 1 to 1.78 (SD 1.44, 95% CI 1.06–2.49) on day 21 with a *p*‐value of 0.02. In contrast, the control group's mean Juniper RQLQ score decreased from 2.39 (SD 1.28, 95% CI 1.71–3.08) on day 1 to 1.69 (SD 0.98, 95% CI 1.16–2.21) on day 21 with a *p*‐value of 0.01. However, there was no significant difference in quality of life between the treated and control groups at the end of the study period (day 21), with *p*‐values of 0.57 for SNOT‐22 and 0.98 for Juniper RQLQ (Figure 4).

### 3.5 Safety

One pre‐menopausal female patient had elevated CTx levels prior to the study (996 ng/L), which normalized at the follow‐up after the trial. All other patients were within the reference interval both before and after the trial.

Adverse events observed in both groups were predominantly mild, with symptoms including sneezing, nasal blockage, nasal drip, and itching in the nose and eyes being the most reported. In the prednisolone‐treated group, one patient experienced mood swings, a stomach flu, and a cold with fever. Additionally, in the control group, one participant reported eye twitching and a period of increased stress and mild depression, while another experienced a transient episode of tachycardia. No SAEs were reported during the study period.

## 4 DISCUSSION

Contrary to our initial hypothesis, the administration of Prednisolone tablets during the 2018 tree pollen season did not yield a significant reduction in symptoms or the usage of topical medication compared with antihistamines. Surprisingly, the group receiving antihistamine tablets exhibited a 4% lower reduction in the dCSMS compared to those treated with prednisolone tablets.

Our intervention strategy was implemented after the initial phase of the tree pollen season, specifically targeting the onset of birch pollen and anticipating the subsequent surge in oak pollen levels. This intervention was timed to coincide with a critical period of heightened tree allergen exposure, with the goal of alleviating symptoms during the latter part of the tree pollen season. By administering medication during the birch pollen period before the anticipated increase in oak pollen levels, we aimed to mitigate symptoms throughout the remainder of the tree pollen season. Using a GAM, we assessed the RR for symptoms in the study group over time, but the model has limitations and the data should be interpreted with caution.

QoL assessments demonstrated improvement in both treatment groups over the study duration, with no discernible difference between the groups at the trial's conclusion. This suggests a general improvement in QoL across participants, likely attributable to the reduced pollen count. Notably, both groups reported symptoms and medication usage above baseline on day 1, potentially due to data collection commencing post‐pollen peak. Importantly, no SAEs were reported, with full compliance observed among participants and high response rates achieved on QoL questionnaires.

Even though no significant differences in dCSMS were obtained between the two treatment groups, the efficacy of prednisolone in alleviating severe symptoms, particularly nasal obstruction, which profoundly impacts patient quality of life and nasal functionality, must be acknowledged. Due to its potent anti‐inflammatory properties, prednisolone is often considered preferable for rapidly relieving severe nasal blockages by significantly reducing mucosal swelling. However, the integration of improvements in nasal obstruction within the overall symptom scores was done in the study, which may have diluted the isolated effects of prednisolone on this specific symptom.

In clinical practice, corticosteroid tablets are commonly administered reactively rather than prophylactically. This approach is especially prevalent during periods when standard treatments fail to provide adequate relief, particularly in anticipation of elevated pollen levels. In Sweden, the tree pollen season spans several months, typically beginning in January/February with the flowering of hazel and alder, reaching its peak with birch in the latter half of April, and concluding with oak by the end of May. Birch pollen, being the most prevalent trigger for AR in Sweden, receives considerable attention in clinical practice, notably due to the availability of Allergen Immunotherapy with Alutard Birch SQ.

During 2018, the birch pollen integral was approximately 50% of the average recorded between 1975 and 2018. Historically, Alutard SQ three‐tree‐pollen, containing birch, oak, beech, hornbeam, hazel, and alder, served as a precursor to Alutard SQ birch. The oak pollen integral during 2018 was approximately 138% of the average recorded between 1975 and 2018. Notably, all patients enrolled in our study exhibited sensitization to both birch and oak pollen.

The timing of treatment in our study mirrored the typical clinical approach to corticosteroid therapy. This approach was predicated on the understanding that such a strategy could offer significant symptom relief when conventional treatments prove inadequate, particularly in the context of sustained pollen exposure.

Questions regarding the optimal treatment regimens for AR with systemic corticosteroids, including considerations of dosage, duration of treatment, and administration methods, remain unanswered. These inquiries are of great importance given that an estimated 10%–20% of the global population suffers from AR and may necessitate systemic corticosteroids at some stage. However, the limited research in this area has led to international guidelines predominantly relying on clinical experience rather than evidence from double‐blinded randomized controlled trials.^2^

Short courses of oral steroids are frequently prescribed in primary care settings, addressing a variety of conditions beyond seasonal AR and asthma, including upper respiratory infections, spine conditions, acute bronchitis, connective tissue disorders, joint disorders, and skin disorders.^16^ Despite the perceived harmlessness of short courses of oral corticosteroids, analyses of private insurance claims involving 1.5 million individuals have revealed significantly elevated rates of sepsis, venous thromboembolism, and fractures among those treated with steroids, even with the relatively brief duration of systemic steroid therapy.^16^ Treating asthma with short courses of corticosteroids in high doses has been linked to the accumulation of higher doses compared with maintenance treatment, potentially resulting in adverse outcomes.^17^ Given the numerous and severe side effects associated with systemic corticosteroids, caution is warranted in their prescription.

This study's design includes crucial limitations that merit attention. Firstly, the modest sample size of 39 participants, though calculated to detect clinically meaningful differences between treatment groups, might not fully capture the variability observed in larger studies. For statistical evaluation, the Mann–Whitney *U* test and the Wilcoxon matched‐pairs signed rank test were employed for all analyses except for one specific analysis detailed in the appendix assessing the RR for symptoms over time within the study group. The use of these non‐parametric tests was deemed appropriate given the sample size and distribution of the data. The use of GAMs is the risk for potentially overfitting or underestimating variability in small samples is a notable concern. The GAM results in the appendix must therefore be interpreted with caution.

In our previous investigation, the administration of methylprednisolone for seasonal AR demonstrated a statistically significant albeit modest divergence from placebo.^9^ While historical studies advocate for the efficacy of systemic steroids in AR management, their clinical significance remains ambiguous. The absence of treatment efficacy observed in our current study may stem from various factors, including dosage regimen, treatment duration, pollen exposure, or potentially underpowered study design. Augmenting systemic steroid dosage or prolonging treatment duration may not necessarily translate into notable alleviation of symptoms and could potentially elevate the risk of adverse effects compared to standard therapeutic modalities.

In conclusion, in alinement with our previous report, the findings of this study suggest that prednisolone tablets did not exhibit superior efficacy over antihistamine tablets in reducing symptoms or medication usage in individuals with tree pollen‐induced AR. Further, the presented findings underscore the importance of prioritizing evidence‐based practices and patient‐centered care in the management of AR. Considering the limited efficacy demonstrated, clinicians should approach the management of seasonal AR with caution. Individualized treatment strategies, tailored to patient preferences and tolerability, remain paramount.

## AUTHOR CONTRIBUTIONS

**Carl Skröder**: Writing—original draft; conceptualization; data curation; project administration; formal analysis; visualization; validation; methodology; investigation. **Laila Hellkvist**: Writing—review and editing; conceptualization. **Ulla Westin**: Conceptualization; writing—review and editing. **Pernilla Sahlstrand‐Johnsson**: Writing—review and editing. **Kerstin Hansson**: Investigation; methodology. **Agneta Karlsson**: Investigation; methodology. **Åslög Dahl**: Investigation; formal analysis. **Leif Bjermer**: Conceptualization; writing—review and editing. **Lars Olaf Cardell**: Conceptualization; investigation; funding acquisition; writing—original draft; methodology; validation; writing—review and editing; project administration; supervision; resources.

## CONFLICT OF INTEREST STATEMENT

The authors declare no conflicts of interest.

## Supporting information

Figure S1

## Data Availability

The data that support the findings of this study are available from the corresponding author upon reasonable request.
