# Supplementary material for: Prednisolone versus antihistamine for allergic rhinitis: No significant difference found in randomized trial
Source: Clin Transl Allergy. 2025 Jan 9;15(1):e70017. doi: 10.1002/clt2.70017 (PMC11717555; doi:10.1002/clt2.70017)
Supplement: Supplementary file 1 — Figure S1 [file CLT2-15-e70017-s001.docx]

Supplement


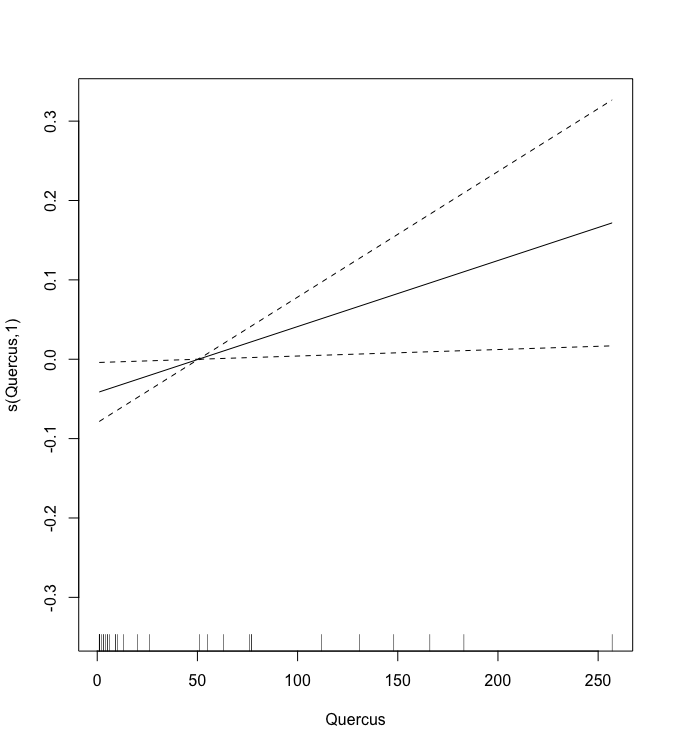


**Figure S1.** The relativ risk (RR) of allergic rhinitis symptoms at different oak pollen concentrations.
